# Expression of an Oncogenic BARD1 Splice Variant Impairs Homologous Recombination and Predicts Response to PARP-1 Inhibitor Therapy in Colon Cancer

**DOI:** 10.1038/srep26273

**Published:** 2016-05-20

**Authors:** Ozkan Ozden, Faraz Bishehsari, Jessica Bauer, Seong-Hoon Park, Arundhati Jana, Seung Hyun Baik, Judith C. Sporn, Jonas J. Staudacher, Cemal Yazici, Nancy Krett, Barbara Jung

**Affiliations:** 1 Department of Medicine, Division of Gastroenterology, University of Illinois at Chicago, Chicago, IL 60612, U.S.A; 2 Department of Internal Medicine, Division of Gastroenterology, Rush University Medical Center, Chicago, IL, 60612, U.S.A; 3 Department of Radiation Oncology, Robert Lurie Cancer Center, Northwestern University Feinberg School of Medicine, Chicago, IL 60611, U.S.A.

## Abstract

BRCA1-associated RING domain protein 1 (BARD1) stabilizes BRCA1 protein by forming a heterodimeric RING-RING complex, and impacts function of BRCA1, including homologous recombination (HR) repair. Although colon cancer cells usually express wild type BRCA1, presence of an oncogenic BARD1 splice variant (SV) in select cancers may render BRCA1 dysfunctional and allow cells to become sensitive to HR targeting therapies. We previously reported association of loss of full-length (FL) BARD1 with poor prognosis in colon cancer as well as expression of various BARD1 SVs with unknown function. Here we show that loss of BARD1 function through the expression of a BARD1 SV, BARD1β, results in a more malignant phenotype with decreased RAD51 foci formation, reduced BRCA1 E3 ubiquitin ligase activity, and decreased nuclear BRCA1 protein localization. BARD1β sensitizes colon cancer cells to poly ADP ribose polymerase 1 (PARP-1) inhibition even in a FL BRCA1 background. These results suggest that expression of BARD1β may serve as a future biomarker to assess suitability of colon cancers for HR targeting with PARP-1 inhibitors in treatment of advanced colon cancer.

Colorectal cancer (CRC) is the third most common cancer worldwide, and in 2014, there were an estimated 50,710 deaths from CRC in the U.S.^1^,^2^. Despite improvements in early detection, over half of the cases are diagnosed at advanced stages which are associated with a poor outcome. Recent data show a trend towards an increase in metastatic cases in young patients, underscoring the need for targeted therapeutic options in advanced disease^3^. Examination of a large cohort of colon carcinomas showed that homologous recombination (HR) repair is deficient in greater than 78% of tumors^4^. Therefore, therapeutics which target HR deficiencies may be effective in colon cancer.

BRCA1 is a tumor suppressor which displays E3 ubiquitin ligase activity and has been associated with HR repair, cell cycle checkpoint regulation, and chromatin dynamics^5^,^6^. BRCA1 is stabilized by forming a heterodimer with BRCA1-associated RING domain protein 1 (BARD1) which binds through their RING finger domains^7^. Dimerization not only stabilizes the two monomers, but is also essential for BRCA1’s tumor suppressive activities^8^,^9^. Therefore, mutations affecting the BARD1/BRCA1 heterodimer structure may confer an increase in cancer risk. Notably, germline mutations in BRCA1 are associated with a predisposition to breast and ovarian cancers^10^,^11^ and, while rare, germline mutations in BARD1 also contribute to hereditary and sporadic breast and ovarian cancer^12^. BRCA1 is usually wild type in sporadic colon cancers, however, germline mutations to BRCA1 have been described to be increased in early onset colon cancers^13^.

HR defective cells, such as breast cancer cells harboring mutant *BRCA1*, display greater than normal sensitivity to poly ADP ribose polymerase 1 (PARP-1) inhibition^14^. Treatment with PARP-1 inhibitors (PARPi) results in an increase in single-strand DNA break formation^15^,^16^,^17^, which if they persist unrepaired, collision of unresolved single strand breaks with replication forks is thought to result in double-stranded breaks (DSB)^18^,^16^. Binding of BARD1-BRCT domain to PAR motifs is important for the early recruitment of BRCA1 to DNA damage sites^19^ and the BARD1/BRCA1 heterodimer plays an important role in the repair of DSB by HR^20^. In addition, BARD1/BRCA1 dimers exhibit ubiquitin ligase activity, which also targets proteins of the RNA polymerase complex and they are subsequently degraded by the proteasome to initiate HR repair^21^. If HR does not function properly and DSBs are not repaired, cell death can occur^14^.

Typically in colon cancer, BRCA1 is wild type, but we have reported that FL BARD1 expression may be lost, which is associated with poorer outcome. Further, various, differentially expressed BARD1 SVs have been reported^7^,^22^,^23^. In colon cancer, we observed a BARD1 SV, BARD1β which lacks exons 2 and 3, encoding most of the RING finger domain^22^. BARD1β has also been reported to be expressed in neuroblastoma, non-small cell lung cancer, breast and ovarian cancers^21^,^22^,^23^,^24^. While FL BARD1 is a tumor suppressor, BARD1β has been reported to be pro-proliferative and tumor initiating^22^,^23^,^24^. Its overexpression leads to neoplastic cell transformation in murine fibroblasts, and its repression leads to growth arrest of cancer cells^22^,^24^,^25^. BARD1β expression has been reported to correlate with poor survival in colon cancer^23^. However, insights into the precise mechanisms of how isoforms may contribute to tumorigenesis in colon cancer and specifically how knowledge of expression may be harnessed in treatment strategies of colon cancer patients are lacking.

In this study, we demonstrate that the expression of the BARD1β correlates with impaired HR in PARPi-sensitive colon cancer cells. Moreover, exogenous expression of the BARD1β isoform in PARPi-resistant colon cancer cells lead to a more transformed phenotype and impaired HR. These cells also display an increased sensitivity to PARPi, suggesting possible therapeutic applications for cancer patients expressing the BARD1β. Treatment with PARPi in conjunction with the DNA-damaging chemotherapeutic agents, Irinotecan or Oxaliplatin, leads to an increased sensitivity to both agents when BARD1β is expressed. We conclude that BARD1β merits further investigation as a therapeutic biomarker for the use of adjunct PARPi therapy in advanced colon cancer.

## Results

### Human colon cancer cells exhibit differential sensitivity to PARP-1 inhibition

**Figure 1:**
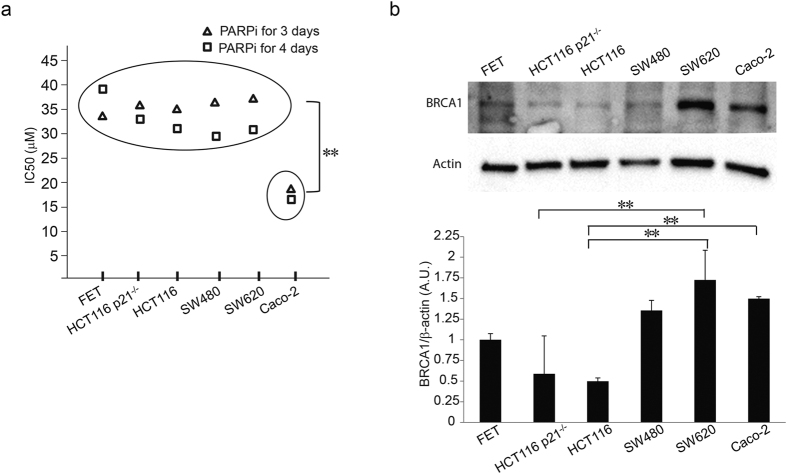
Human colon cancer cells exhibit differential sensitivity to PARP-1 inhibition. (**a**) CRC cell lines FET, HCT116 p21^−/−^, HCT-116, SW480, SW620, and Caco-2 were treated with PARPi for 3 (triangle) and 4 (square) days and IC_50_ determined (**p < 0.01). The experiment was performed at least three independent trials with at least three technical replicates. (**b**) Untreated CRC cell lines were harvested, protein extracts were prepared, separated and immunoblotted with anti-BRCA1 antibody as described in Methods. The experiment was repeated at least three times. Quantification of protein band intensity was performed by ImageJ software (*p < 0.05 and **p < 0.01).

Increased sensitivity to PARP-1 inhibition is indicative of defective DNA double-strand break repair by homologous recombination^16^,^26^. To determine etiologies of PARPi sensitivities in colon cancer cells, we treated a panel of colon cancer cell lines representing a spectrum of genomic backgrounds with increasing concentrations of PARPi (Fig. 1a). Cell viability and dose response curve for each cell line was determined after 3 and 4 days of treatment and IC_50_s were calculated. None of the known genomic determinants of colon carcinogenesis, including microsatellite instability (MS), loss of SMAD4 or loss of p21, were associated with an increased sensitivity to PARPi. Yet the MS stable Caco-2 cell line was significantly more sensitive to PARPi, suggestive of an undefined distinct molecular feature in these cells (Table 1).

### Differential sensitivity to PARPi among colon cancer cell lines is not associated with BRCA1 mutation or loss of BRCA1 expression

BRCA1 is a major participant in HR, therefore cells deficient in BRCA1 show increased lethality with PARPi due to absence of DSB DNA repair by HR. All tested colon cancer cell lines are known to have wild type *BRCA1* and all express FL BRCA1 protein. BRCA1 protein was in similar quantities among SW620, Caco-2, and SW480 cells and these were relatively higher than FET, HCT116 p21^−/−^, HCT116 (Fig. 1b).

**Figure 2:**
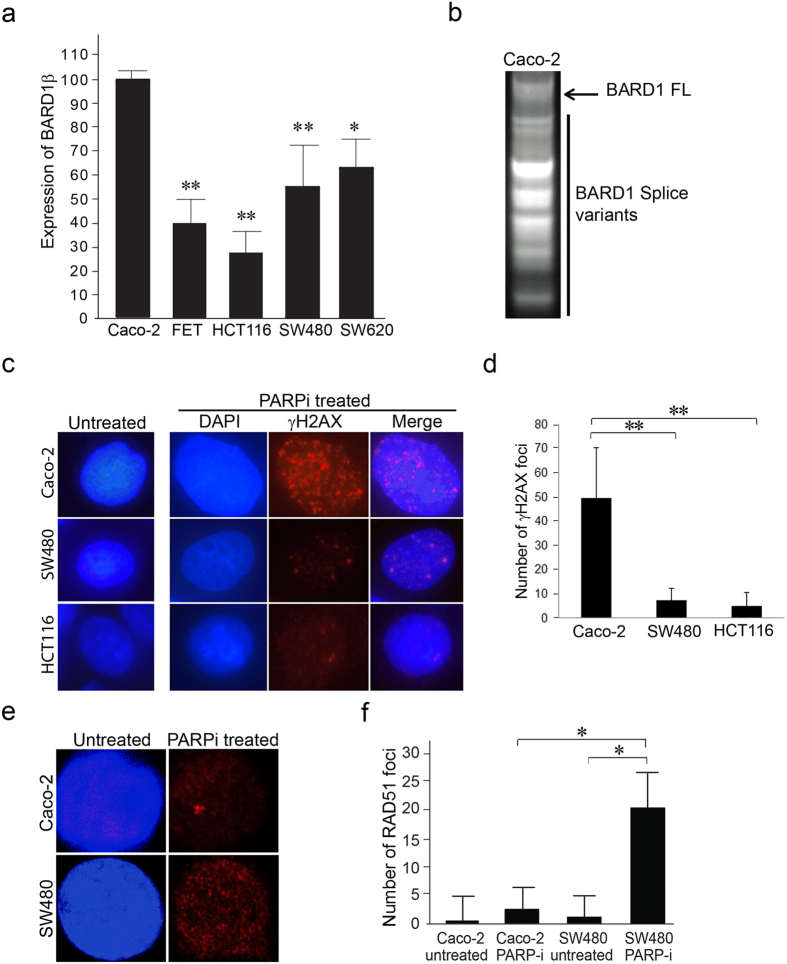
Differential sensitivity to PARP inhibition among colon cancer cell lines is not associated with BRCA1 mutation or loss of BRCA1 expression. (**a**) Fold change in BARD1β among select colon cancer cell lines were compared using SYBR green real-time PCR and reported as a percentage of the expression level of PARPi sensitive colon cancer cell line, Caco-2. Quantitative PCR was performed at least three times from three different RNA extractions for each cell line. (*p < 0.05 and **p < 0.01) (**b**) Real-time PCR on polysomal fractions of Caco-2 colon cancer cells showed amplification of various SVs of BARD1. (**c**) Untreated and 10 μM PARPi treated Caco-2 and SW480 cells for 4 days were stained with both 4′,6-diamidino-2-phenylindole (DAPI) nuclear fluorescent stain and phospho-histone γH2AX DSB marker. Scale bar represents 5 μm. (**d**) Nuclear phospho-histone γH2AX foci formation in Fig. 3c were quantified (*p < 0.05 and **p < 0.01). (**e**) Untreated Caco-2 and SW480 cells were stained with DAPI and anti-RAD51 antibody. 10 μM PARPi treated Caco-2 and SW480 cells for 4 days were stained with DAPI and anti-RAD51 antibody. (**f**) Nuclear RAD51 foci formation in Fig. 2e were quantified (*p < 0.05 and **p < 0.01).

Alterations in BARD1 may affect BRCA1 function in a *BRCA1* wild type setting. FL BARD1 is present in both PARPi-sensitive and -resistant cell lines. However, BARD1 SVs may have functions independent from its FL protein and may affect the function of BRCA1 independent of their effects on BARD1 expression. BARD1β has been reported to have poor prognosis in colon cancer^22^. Using real-time PCR, we compared the expression of BARD1β in a panel of colon cancer cell lines. Expression of BARD1β was significantly higher in PARPi sensitive Caco-2 cells compared to its expression in cell lines that were resistant to PARPi (Fig. 2a).

### BARD1 SV mRNA is associated with polysomes

To assess potential functions of the BARD1β, we determined whether the SVs mRNAs are translated. Real-time PCR of cDNA with primers targeting the first and last BARD1 exon revealed amplification of multiple SVs in the polysomal fractions, which is congruent with active translation (Fig. 2b).

### PARPi causes DSBs in both sensitive Caco-2 and resistant SW480 cells

PARP-1 activity is crucial in the recognition and repair of DNA single-strand breaks. Blocking PARP activity with the PARP-1 inhibitor ABT888 causes single strand breaks to accumulate, leading to formation of double-strand breaks^27^,^16^,^14^. We compared the formation of double-strand breaks in the PARPi-sensitive Caco-2 cells and the PARPi-resistant SW480 and HCT116 cells, using γH2AX foci formation as an indicator (Fig. 2c). Untreated control cells displayed very few or no γH2AX foci formation, consistent with the absence of double-strand breaks, while treatment with the PARPi induced double-strand breaks in both PARPi-sensitive (Caco-2) and PARPi-resistant cells (SW480 and HCT116). PARPi caused significantly more γH2AX staining in the Caco-2 cells (Fig. 2d). This confirms PARPi causes double-strand breaks both in the PARPi-sensitive and PARPi-resistant cell lines further focusing on differences in DNA repair mechanism as the basis for PARPi sensitivity.

### PARPi-sensitive colon cancer cells show impaired HR

We hypothesized that BARD1β expression in the PARPi-sensitive colon cancer cells impacts HR proficiency, which can be measured by the formation of RAD51 foci. RAD51 is a critical protein functioning in the HR DNA repair, which localizes to the nucleus to form foci upon DNA damage, and are detected by a florescent antibody and counted using a fluorescent microscope^15^. We determined the RAD51 foci formation in the PARPi-sensitive and -resistant colon cancer cells following treatment with PARPi. Each cell line was treated with a concentration of PARPi lower than its respective IC_50_ (10 μM) to avoid lethality. As expected, there were very few or no foci formed in untreated cells (Fig. 2e, left panels; 2f). After treatment with PARPi, PARPi-resistant cells (SW480) (Fig. 2e, right lower panel; 2f) displayed increased RAD51 foci formation compared to PARPi-sensitive colon cancer cells (Caco-2) which displayed a significantly weaker HR DNA repair response characterized by lower RAD51 foci formation (Fig. 2e, right upper panel; 2f). Therefore, impaired HR function may be due to BRCA1/BARD1 deficiency in a *BRCA1* wild type setting.

### Exogenous expression of SV BARD1β in PARPi-resistant cells results in impaired HR

**Figure 3:**
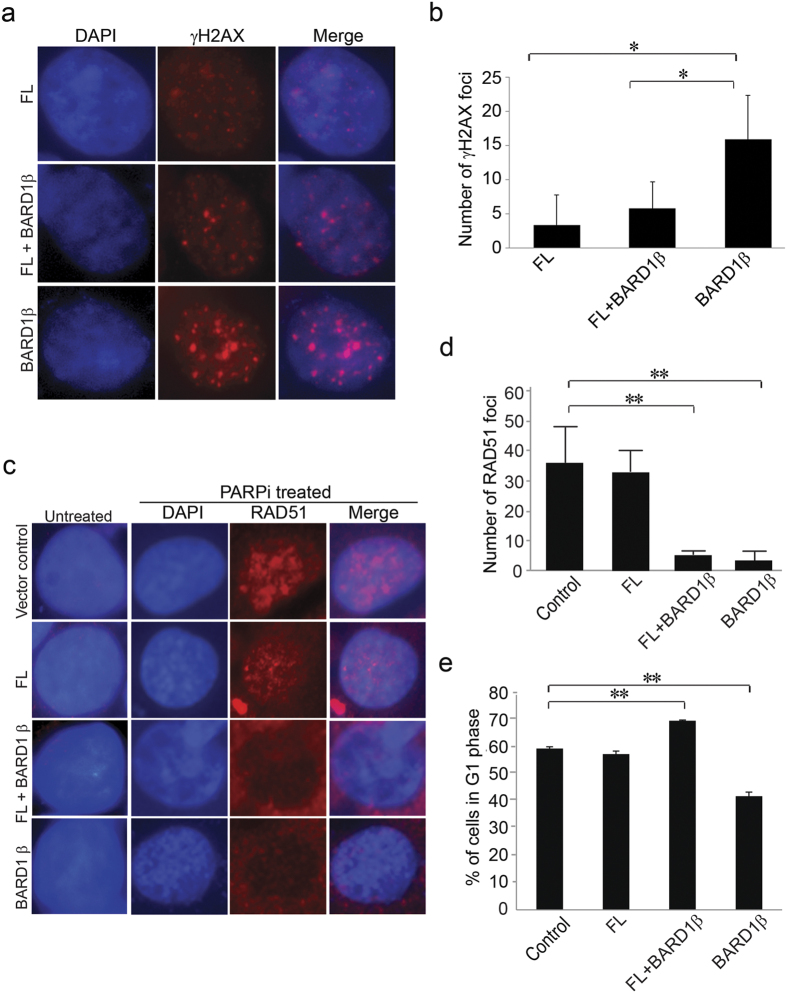
PARPi-sensitive colon cancer cells show impaired HR. (**a**) FL BARD1 expressing SW480 cells, both FL and BARD1β expressing SW480 cells, and BARD1β expressing SW480 cells were exposed to PARPi for 72 hours and stained with DAPI and phospho-histone γH2AX DSB marker. Stably infected SW480 cells were treated with PARPi, and then fixed and stained with DAPI and phospho-histone γH2AX antibody. (**b**) Nuclear γH2AX foci formation in Fig. 3a were quantified (*p < 0.05 and **p < 0.01). (**c**) Control SW480 cells, FL BARD1 expressing SW480 cells, both FL and BARD1β expressing SW480 cells, and BARD1β expressing SW480 cells were stained with DAPI and anti-RAD51 antibody. Stably infected SW480 cells were treated with 10 μM PARPi for 4 days, and then fixed and stained with DAPI and anti-RAD51 antibody. (**d**) Nuclear RAD51 foci formation in Fig. 3c were quantified (*p < 0.05 and **p < 0.01). (**e**) Control and FL, FL + BARD1β and BARD1β expressing SW480 cells were stained with PI, and monitored by flow cytometry to determine the percentage of G1 phase cells. Experiments were repeated at least three times (*p < 0.05 and **p < 0.01 relative to control).

To test the hypothesis that expression of BARD1β leads to impaired HR, we expressed BARD1β in the SW480 PARPi-resistant cells, which have relatively low levels of endogenous BARD1β transcript and have very similar levels of BRCA1 as Caco-2 PARPi-sensitive cells have. Additionally, as reference controls, stable SW480 cells expressing V5 tagged FL BARD1, or both BARD1β and FL BARD1, or an empty vector were prepared (Supplementary Fig. S1). Although HCT116 cells had considerably lower levels of endogenous BARD1β transcript relative to SW480 cells, we decided to prepare stable SW480 cells expressing BARD1β because HCT116 cells have significantly lower levels of endogenous BRCA1 protein, and are deficient in DNA mismatch repair. Exogenous overexpression of BARD1β in SW480 cells caused significantly more γH2AX foci than FL + BARD1β cells in response to PARPi treatment (Fig. 3a,b) indicating increased sensitivity to PARPi.

To evaluate the effect of BARD1β on HR function, RAD51 foci formation was compared among the PARPi-resistant SW480 colon cancer cell lines: SW480 expressing BARD1β, FL BARD1, or both BARD1β and FL BARD1 with and without PARPi treatment (Fig. 3c). In the absence of PARPi, no or only minimal RAD51 foci formation was observed in all cell lines (Fig. 3c, left panels; 3d). However, when the cells were exposed to PARPi, control and FL BARD1 expressing cells displayed significantly higher accumulation of RAD51 (Fig. 3c, left upper three panels; 3d), while both BARD1β expressing and the combined BARD1β and FL BARD1 expressing cells exhibited significantly fewer foci (Fig. 3c, left lower three panels; 3d) consistent with loss of BRCA1/BARD1 function with expression of BARD1β independent of FL BARD1 expression. The reduction of RAD51 foci formation in BARD1β expressing cells does not stem from arrest of cells in G1 phase (Fig. 3e) and (Supplementary Fig. S2). Cell cycle analysis showing no accumulation of cells in G1 phase supports the idea of HR deficiency.

### Expression of BARD1β in PARPi-resistant cells increases cytoplasmic localized BRCA1

**Figure 4:**
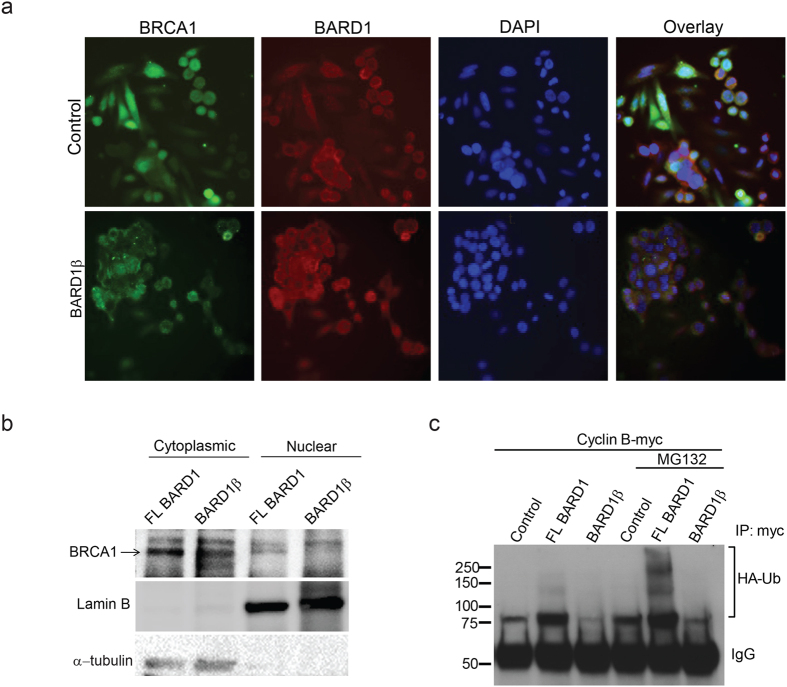
Expression of BARD1β in PARPi-resistant cells increases cytoplasmic localized BRCA1 and decreased ubiquitin ligase activity of BRCA1. (**a**) Control and BARD1β expressing SW480 cells were incubated with anti-BARD1 (red) and anti-BRCA1 (green) for 16 h following fluorescence secondary antibody staining. Nuclei were stained with DAPI. (**b**) Nuclear and cytoplasmic compartments were isolated by fractionation of stable FL BARD1 and BARD1β expressing SW480 cells. Immunoblotting for BRCA1 is indicated in the top panel, lamin B (control for nuclear fraction) and α-tubulin (control for cytoplasmic fraction) are in the middle and bottom panels respectively. (**c**) Stable control, full length BARD1 expressing and BARD1β expressing SW480 cells transfected with ubiquitin-FLAG and cyclin B-MYC, and treated with MG132. Detection of ubiquitin-FLAG or cyclin B-MYC was carried by immunoprecipitation with an antibody against MYC followed by immunoblot with an antibody against FLAG-tag.

BARD1 plays an important nuclear chaperone role for BRCA1^28^,^29^,^30^,^31^,^32^,^33^,^34^, suggesting that dimerization of BRCA1 with BARD1 is required for nuclear localization of BRCA1. Since BARD1β is missing most of the dimerization region, we examined the subcellular localization of BRCA1 in control and BARD1β stably expressing PARPi-resistant cells using immunofluorescence (Fig. 4a). In control or FL BARD1 overexpressing PARPi-resistant cells, BCRA1 was mainly localized to the nucleus, however, when BARD1β was overexpressed, we observed BRCA1, both, in the cytoplasm and nucleus implying BARD1β is deficient in nuclear chaperone activity (Fig. 4a). Cell fractionation to separate the nuclear and cytoplasmic fractions was performed to confirm differential localization of BRCA1 between FL and BARD1β expressing cells. FL BARD1 expressing cells displayed greater amount of nuclear BRCA1 than BARD1β expressing cells (Fig. 4b).

### Impact of BARD1β on ubiquitin ligase activity of BRCA1

Mutations within the RING binding domain leads to impairment of E3 ubiquitin ligase activities of BRCA1^8^,^29^,^30^,^31^. BRCA1 has been reported to ubiquitinate cell cycle proteins including cyclin B; which ultimately contributes to regulation of G2/M cell cycle checkpoint^6^. We examined the impact of BARD1β expression in PARPi-resistant cells on E3 ubiquitin ligase activity of BRCA1, and ubiquitination of cyclin B. PARPi-resistant cells were co-transfected with His-tagged ubiquitin and MYC tagged cyclin B and treated with MG132, an inhibitor of the proteasome to reduce degradation of ubiquitinated molecules. Consistent with functional BARD1 in control and FL BARD1 overexpressing cells, we detected relatively high ubiquitination activity. In contrast, cells expressing BARD1β displayed little or no ubiquitinated cyclin B following MG132 treatment (Fig. 4c). Taken together, these findings suggest that colon cancer cells which express BARD1β display decreased ubiquitination and are more sensitive to PARPi due to impaired HR.

### Expression of BARD1β is associated with a more invasive phenotype

**Figure 5:**
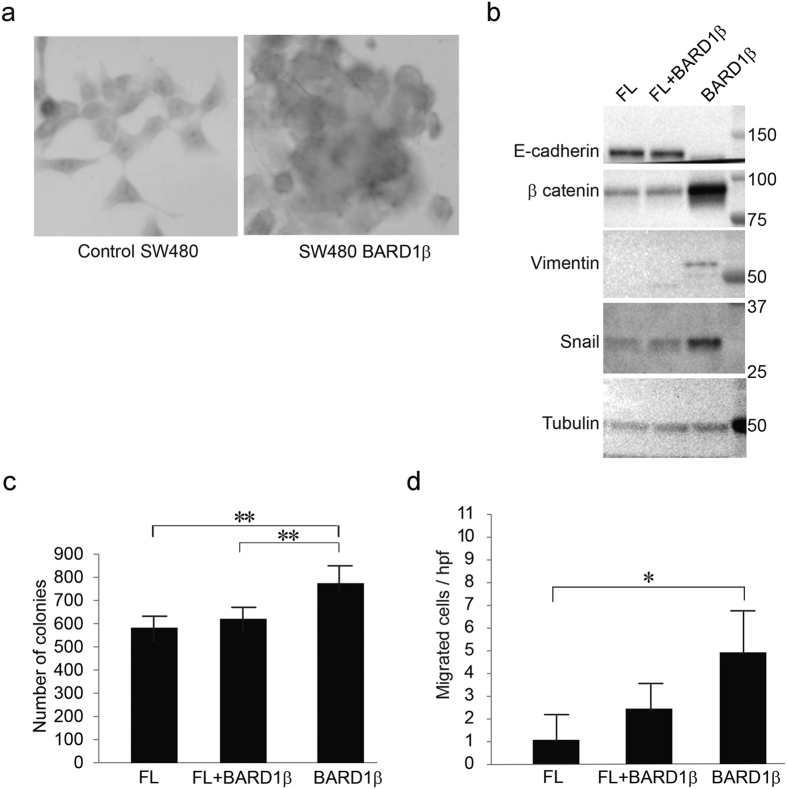
Expression of BARD1β is associated with a more invasive phenotype. (**a**) Control (left) and BARD1β expressing (right) SW480 cells were stained with hemotoxylin, and inspected under a light microscope (400x magnification). (**b**) Stable SW480 cells infected with either FL BARD1, BARD1β or the combination (FL + BARD1β) were harvested, separated, and immunoblotted for EMT proteins: E-cadherin, β-catenin, vimentin, or snail. Tubulin was used as a loading control. (**c**) Colony formation assay of stable SW480 cells: 100 cells were seeded into six-well plates and incubated for two weeks. Colonies were fixed and stained with crystal violet (*p < 0.05 and **p < 0.01). (**d**) The relative number of migrated stable SW480 cells per high power field (hpf) (*p < 0.05 and **p < 0.01).

We have previously observed that expression of BARD1β is associated with poor prognosis in colon cancer tumors^22^. To test if expression of BARD1β leads to a more transformed phenotype, we examined the differences in histology when BARD1β is over-expressed in PARPi-resistant cells (Fig. 5a). Under standard cell culture conditions, control and FL BARD1 expressing PARPi-resistant cells formed single cell monolayers, while BARD1β expressing cells formed clusters indicative of loss of cell-cell adhesion. The transition to metastatic disease includes epithelial to mesenchymal transitions (EMT) and is associated with an increase in cellular ability to form colonies. We examined BARD1β overexpressing PARPi-resistant cells for expression of EMT marker proteins by immunoblotting, and observed a decrease in E-cadherin and increases in β-catenin, Vimentin and Snail, all consistent with induction of EMT (Fig. 5b).

Next, we compared the clonogenic capacity of PARPi-resistant cells expressing either FL BARD1 or BARD1β, or both using the colony formation assay as previously described^32^. BARD1β expressing cells produced significantly higher numbers of colonies relative to cells expressing either FL or both FL BARD1 and BARD1β, indicating that BARD1β expressing cells retain a higher capacity to produce colonies (Fig. 5c). Increase in migration of BARD1β expressing cells further supports the notion of enhanced tumorigenesis (Fig. 5d).

### Exogenous expression of SV BARD1β in PARPi-resistant colon cancer cells imparts sensitivity to PARP inhibition

**Figure 6:**
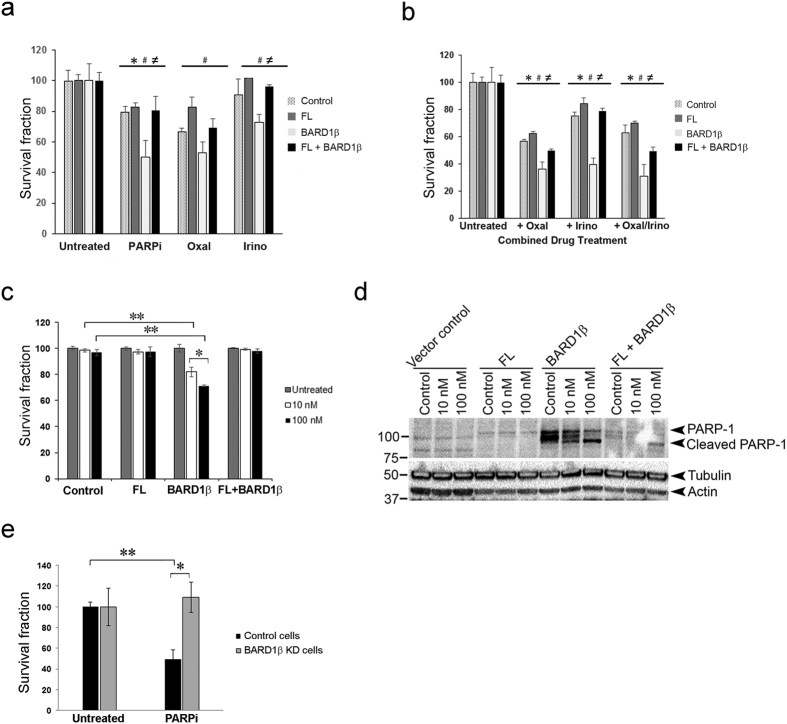
Exogenous expression of SV BARD1β in resistant SW480 cells imparts sensitivity to PARPi. (**a**) SW480 cells stably infected with either FL BARD1, BARD1β, or the combination FL + BARD1β, were exposed to either PARPi (20 μM) for 96 hours, or only Oxaliplatin (50 μM) for 36 hours, or only Irinotecan (5 μM) for 36 hours. Survival fraction was determined using cell viability assays (*control vs. BARD1β; ^#^FL vs. BARD1β; ^≠^BARD1β vs. FL + BARD1β; ^*,#,≠^p < 0.01). (**b**) Stable SW480 cells which were prepared as explained above were exposed to PARPi (20 μM) for 84 h followed by either Oxaliplatin (50 μM) or Irinotecan (5 μM) or both supplementation for 36 h. Survival fraction was determined using cell viability assays (*control vs. BARD1β; ^#^FL vs. BARD1β; ^≠^BARD1β vs. FL + BARD1β; ^*,#,≠^p < 0.01). (**c**) SW480 cells stably infected with either FL BARD1, BARD1β, or the combination FL + BARD1β, were exposed to Olaparib PARPi (10 nM or 100 nM) for 72 hours. Survival fraction was determined using cell viability assays (*p < 0.05 and **p < 0.01). (**d**) Protein lysates (40 μg) from each sub-line of SW480 cells were separated and immunoblotted with anti-PARP1 antibody as described in Methods. For normalization, blots were probed with α-tubulin and β-actin. This blot is representative of three independent experiments. (**e**) shRNAs targeting BARD1β were prepared, and BARD1β was stably knocked down in Caco-2 cells as described in Methods. Cells were exposed to 10 μM ABT-888 for 72 hours. Survival fraction was determined using cell viability assays (*p < 0.05 and **p < 0.01).

To evaluate the effect of BARD1β expression on PARPi-mediated cell death, we exposed stable transfectants of PARPi-resistant cells, characterized above, to low dose PARPi. Compared to control and FL BARD1 expressing cells, expression of BARD1β sensitized cells to low concentrations of PARPi (Fig. 6a). When FL BARD1 and BARD1β are co-expressed, we observed an intermediate phenotype with respect to PARPi sensitization (Fig. 6a).

DNA damaging agents including Irinotecan and Oxaliplatin are commonly used in the treatment of metastatic colorectal cancers^33^. Irinotecan is a topoisomerase 1 poison leading to the formation of a potentially lethal DNA double-stranded break. Oxaliplatin, in contrast, is a platinum analogue, which causes the formation of inter- and intra-strand crosslinks in DNA that prevent transcription and replication. Ultimately this results in DNA damage and cell death. We investigated if PARPi can sensitize colon cancer cells expressing BARD1β *in vitro* to either Oxaliplatin and/or Irinotecan treatment. We established the optimal time of drug exposure and observed that Irinotecan and Oxaliplatin work more rapidly and achieve optimal action at 36 hours while PARPi requires 96 hours of incubation (Supplementary Fig. S3). Similarly we established concentrations for each drug that are sub-lethal to allow for optimal action when used in combination (Supplementary Fig. S3). Expression of BARD1β in PARPi-resistant cells did not selectively sensitize the cells to either Oxaliplatin or Irinotecan alone relative to control cells (Fig. 6a). Due to the differential time course of drug response, we chose to treat cells sequentially, first with PARPi followed by Oxaliplatin or Irinotecan. Addition of Oxaliplatin or Irinotecan decreased the fraction of viable cells in all stable PARPi-resistant cells (Fig. 6b); however, addition of Irinotecan to PARPi pre-treated cells created higher specificity with respect to killing of BARD1β expressing PARPi-resistant cells. These data indicate that colon cancer patients with high expression of BARD1β may receive therapeutic benefit from a serial combination of PARP inhibition and Irinotecan treatment.

To test whether BARD1β expressing SW480 cells was exclusively sensitive to ABT-888 or other PARP inhibitors display a similar feature, we used a potent PARP inhibitor, Olaparib (AZD2281), which has the additional cytotoxic function due to its higher ability of PARP-trapping on chromatin. Consistent to our findings with ABT-888, BARD1β expression in SW480 cells increased sensitivity to Olaparib (Fig. 6c) as well.

In addition to these survival assays, we evaluated whether exogenous expression of BARD1β in SW480 cells increased the overall apoptotic index. Expression of BARD1β increased PARP1 protein expression. Moreover, cleavage of PARP1 was further stimulated after 10 nM and 100 nM treatments of Olaparib which was an indication of increased apoptosis (Fig. 6d).

To further confirm the expression of BARD1β is associated with PARPi sensitivity, we knocked down the expression of BARD1β in PARPi sensitive Caco-2 cells using shRNA-mediated knockdown. Knockdown of BARD1β in Caco-2 cells rendered these cells less sensitive to PARPi relative to control Caco-2 cells (Fig. 6e).

In summary, we show that colon cancer cells which express BARD1β SV display impaired HR DNA repair and are more sensitive to PARP-1 inhibition. Overexpression of BARD1β in PARPi-resistant colon cancer cells rendered these cells sensitive to PARPi, and decreased E3 ligase activity as well as nuclear localization of BRCA1. This was associated with an increase in EMT, as well as colony formation and enhanced cell migration, supporting that oncogenic properties of BARD1β in colon cancer are at least in part, via targeting BRCA1 function.

## Discussion

The tumor suppressor functions of BRCA1 are largely attributed to stabilization of BRCA1 by heterodimer formation with binding partners such as BARD1 within the BRCA1-associated RING domain protein. About twenty percent of clinically relevant BRCA1 mutations occur within the binding site of BRCA1 and BARD1, indicating that the binding of BARD1 is crucial for HR DNA repair function of BRCA1^5^,^34^. BRCA1 is typically wild type in colon cancer, but the presence of various BARD1 SVs of mostly unclear function has been reported^7^,^22^,^23^.

The loss of BARD1 in mice leads to embryonic lethal phenotype and chromosomal instability^9^,^35^. BARD1 spans 11 exons and we previously observed 19 distinct BARD1 SVs in colon cancer patients that account for over 40 percent of the total BARD1 transcript^22^. The individual functions of these SVs have not been assessed. However, BARD1β may have oncogenic activities. In cervical, breast, ovarian and endometrial cancer cell lines, BARD1 SVs missing the RING domain have been reported to be more abundant than the full length protein, and the knockdown of both FL and SVs decreased the rate of proliferation and led to growth arrest^25^.

**Figure 7:**
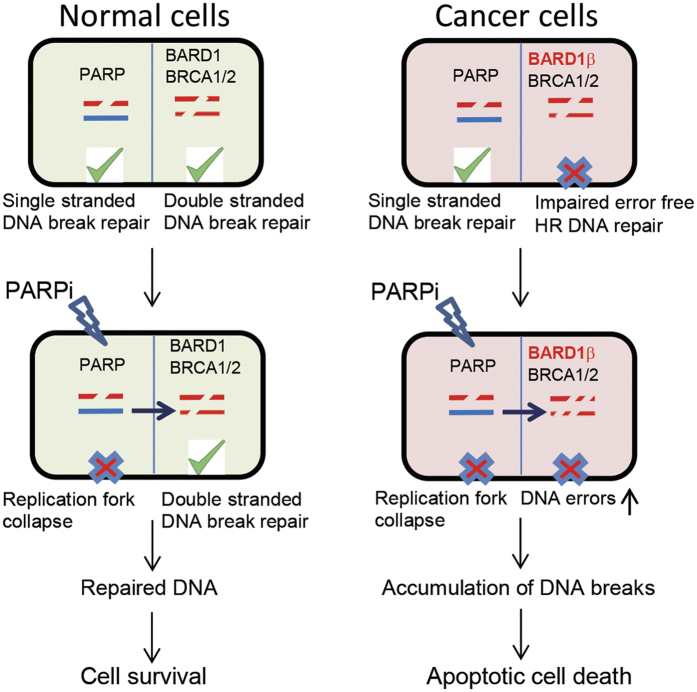
Schematic of sensitization of colon cancer cells to PARPi via BARD1β expression. In normal cells, single strand DNA breaks are repaired via PARP-1 while double strand DNA breaks are repaired via BRCA1/BARD1 dependent HR repair. PARPi treatment of normal cells leads to replication fork collapse and persistence of single strand DNA breaks. However, single strand breaks are converted to double strand DNA breaks by replication and then repaired by HR resulting in cell survival. Therefore, HR DNA repair proficient normal cells are resistant to PARP-1 inhibition. In contrast, overexpression of BARD1β decreases the rate of error-free HR of double strand DNA breaks. HR DNA repair deficiency renders these tumor cells hypersensitive to PARPi leading to apoptosis.

Deficiencies in HR DNA repair sensitize cancer cells to PARPi^36^. PARPi leads to the accumulation of single-strand DNA breaks which are converted to double-strand DNA breaks. In normal cells, PARPi alone is not sufficient to induce apoptosis because the HR DNA repair mechanism repairs double-strand DNA breaks (Fig. 7) leading to cell survival. However, in cancer cells with HR deficiency, other error prone DNA repair mechanisms dominate which increases DNA errors and genomic instability, and ultimately induces apoptosis^14^. We found that BRCA1 protein expression in SW620, Caco-2, and SW480 cells was similar; however, Caco-2 cells had significantly higher levels of BARD1β transcript. This observation supports the idea of BARD1β expression level is a marker for PARPi sensitivity. Overexpression of BARD1β in colon cancer cell lines, such as in Caco-2 cells, impairs error free HR DNA repair by negatively influencing functions of BRCA1 thus sensitizing cells to PARPi. The high numbers of DNA breaks caused by HR DNA repair deficiency coupled with PARP inhibition leads to massive genomic instability resulting in apoptotic cancer cell death (Fig. 7). This increased sensitivity to the PARPi, suggests therapeutic applications for cancer patients expressing this BARD1 isoform. An effective *ex vivo* assay to determine the proficiency of HR in colon cancer patients will be useful for predicting which patient might obtain the most benefit from the therapeutic use of PARPi. Response to PARPi therapy could be predicted using the *ex vivo* RAD51 foci formation assay^37^. This would ultimately provide a biologic basis for therapeutic strategies such as PARPi alone or combination of PARPi with DNA damaging agents in the treatment of colon cancer patients.

In this study, we demonstrate that overexpression of BARD1β stimulated a decrease in BRCA1 functional capabilities with decreased ubiquitination, decreased nuclear translocation, and decreased HR repair. These results indicate that expression of BARD1 SVs lacking the BRCA1 binding domain could predispose patients to colon cancer similar to the predisposition for breast cancer by *BRCA1* mutations. We have previously reported that BARD1β is overexpressed in a subset of colorectal cancer patients. Due to the lack of the ring binding domain, BARD1β does not contain the binding site necessary for interaction with BRCA1 and may display oncogenic effect via affecting BARD1/BRCA1 tumor suppressor function either independent or dependent on FL BARD1 protein. For example, Ryser *et al*. report that FL BARD1 binds to BRCA1 leading to ubiquitination and degradation of Aurora B during mitosis. In contrast BARD1 SV can bind the Aurora B/BRCA2 complex stabilizing Aurora B leading to enhanced cell proliferation^38^. Additional binding partners, such as FL BARD1 and/or PARP, may exist for this BARD1 isoform which contribute to its oncogenic effect. We found that BARD1β expressing SW480 cells had higher levels of PARP1 protein expression relative to FL or both FL + BARD1β expressing SW480 cells. It has been previously reported that PARP1 also has a role in repair of double strand breaks, such as non-homologous end joining (NHEJ) and microhomology-mediated end joining (MMEJ), which are error prone DNA repair pathways^39^,^40^, therefore overexpression of PARP1 in BARD1β expressing cells might also increase genomic stability and cancer.

It has been previously reported that while overexpression of BARD1 SVs results in cell transformation, its repression leads to growth arrest of cancer cells^25^. The exogenous expression of BARD1β isoform in PARPi-resistant SW480 colon cells leads to a more transformed phenotype with enhanced EMT. E-cadherin, a mediator of cell-cell adhesion^41^, is reduced by BARD1β overexpression which can support invasion and growth of tumor cells. In contrast, β-catenin expression was markedly higher in BARD1β expressing cells. β-catenin overexpression has been linked to many epithelial cancers, including colorectal cancer^42^. BARD1β expressing cells exhibited increased levels of the EMT regulator, SNAIL, and mesenchymal marker, vimentin, which could contribute to the observed histological difference between control and BARD1β expressing cells.

In conclusion, BARD1β displays oncogenic activities through negatively affecting the tumor suppressor functions of BRCA1. Our findings indicate therapeutic targeting of deficient HR with PARPi therapy may be beneficial to the colon cancer patients who express this SV. Future development of fast, novel detection methods to identify colon cancer patients with HR deficiency is needed and detection of BARD1β may provide direction.

## Methods

### Chemicals

Aqueous solutions were prepared of ABT888 (sc-202901, Santa Cruz Biotechnology, Dallas, TX), stock concentration of 2 mM; Oxaliplatin (O9512, Sigma, St. Louis, MO), stock concentration of 10 mM; Irinotecan (I1406, Sigma), stock concentration of 10 mM; and Olaparib (10621, Cayman, Ann Arbor, MI), stock concentration of 10 mM.

### Cell culture and generation of stable cell lines

SW480 and Caco-2 cells (ATCC, Manassas, VA, USA) were maintained in 10% Dulbecco’s modified Eagle medium (DMEM), supplemented with 10% fetal bovine serum and 1% antibiotic-antimycotic agent in a 37 °C incubator with 5% CO_2_. Cells were validated by 9 STR (short tandem repeat) profiling, using CellCheck 9 Plus, tested for mycoplasma, and found to be mycoplasma free (both assays performed by IDEXX, Columbia, MO) in February 2015. BARD1β and FL BARD1-expressing lentivirus were constructed in pCDH-CMV-MCS-EF1-Puro cDNA cloning and expression vector (CD510B-1, System Biosciences, San Francisco, CA, USA). SW480 cells stably overexpressing BARD1β, FL BARD1, or both BARD1β and FL BARD1 were generated using lentivirus infection.

### Immunoblotting

Immunoblotting was performed using the Trans-blot Turbo transfer system (Bio-Rad, Hercules, CA) using PVDF membranes. Membranes were then incubated with anti-BRCA1 (Abcam, Cambidge, MA), Flag antibody (Sigma-Aldrich, St. Louis, MO), V5 tag, E-cadherin, snail, vimentin, or β-catenin (Cell Signaling, Danvers, MA), cleaved PARP1 (Santa Cruz, Dallas, TX) primary antibodies for 16 hr at 4 ^o^C.

### Immunofluorescence

Cells seeded on glass coverslips were fixed in 4% paraformaldehyde, followed by blocking with 3% BSA in PBS. Then, cells were incubated with anti-RAD51 (Santa Cruz), anti-P-histone H2AX (Cell Signaling), anti-BRCA1 (Abcam), or anti-BARD1 (Santa Cruz) antibody in PBS, followed by incubation with goat anti-rabbit and anti-mouse IgG conjugated with Alexa Fluor 488 and 594 (Invitrogen) in PBS. Cells were washed in PBS, mounted, and then imaged with a fluorescence microscope (Nikon Eclipse E600 microscope, SPOT Imaging Software, Diagnostic Instruments, MI). γH2AX and RAD51 immunofluorescence assays were performed as previously described^36^,^43^,^44^. An average of 100 nuclei were counted for γH2AX and RAD51 immunofluorescence assays, and experiments performed in triplicates.

### Measurement of proliferation rates and cell survival

FET, HCT116 p21^−/−^, HCT116, SW480, SW620 and Caco-2 cells were seeded into 96-well plate at a concentration of 20,000 cells with 100 μL/well DMEM supplemented with 10% fetal bovine serum and 1% antibiotic-antimycotic agent in a 37 °C incubator with 5% CO_2_ and incubated for 24 hr. A range of concentrations (0, 1, 5, 10, 20, 40 μM) of PARPi were added to the seeded cells and incubated for 72 or 96 hr. Cell proliferation and cell survival were determined according to manufacturer’s instructions using either Cell Counting Kit-8 from Dojindo Laboratories (Rockville, MD) and/or the BioRad automated cell counter (Bio-Rad). The half maximal inhibitory concentrations (IC_50_) of PARPi were determined by constructing a dose-response curve for each cell line. The experiment was performed at least three independent trials with at least three technical replicates.

### Migration assay

Migration assays were performed as previously described^45^ using transwell 12 well plates with 8 μm pores (Corning, Tewksbury, MA) coated with fibronectin (Sigma). Data are presented as the number of migrated cells per high power field (hpf) as the mean +/− s.d. of triplicate assays.

### Ubiquitination assay

E3 ubiquitination ligase activity of BARD1 proteins were compared in control, FL BARD1, and BARD1β expressing SW480 cells as previously described^46^. These cells were co-transfected with HA-tagged ubiquitin and MYC-tagged cyclin B using Fugene HD in the presence and absence of 10 μM proteasome inhibitor MG132 for 3 hr. After 48 hr, Myc-tagged cyclin B proteins were immunoprecitated with anti-Myc antibody, and resolved in 10% SDS PAGE. PVDF membrane was immunoblotted with HA antibody.

### Polysomal fraction assay

To confirm translation of BARD1 SVs, we isolated polysomal fractions from Caco-2 cells. Cells were treated with 0.1 mg/ml cycloheximide (CHX) for 3 min at 37 °C, and harvested into hypotonic lysis buffer [0.5% Triton X-100, 0.5% sodium deoxycholate, 2.5 mM MgCl_2_, 5 mM Tris (pH 7.5), 1.5mM KCl, 0.1 mg/ml CHX, 100 units RNAse-Inhibitor (Ambion-Life Technologies, Grand Island, NY)] at 4 °C. Nuclei were removed, 200 μg/ml heparin was added and residual debris was removed by centrifugation. The lysate was layered on a 10 ml continuous sucrose gradient (10–50% sucrose in 5 mM MgCl_2_, 20 mM HEPES (pH 7.6), 100 mM KCl). After 110 min of centrifugation at 35.000 rpm at 4 °C, the absorbance at 254 nm was measured continuously as a function of gradient depth using the Teledyne Isco UA-6 Absorbance Detector. Prepared polysomal fractions were pooled, and total RNA from polysomal fractions was isolated with the Allprep RNA/Protein Kit from Qiagen (Hilden, Germany). Reverse transcription-PCR of isolated RNA was performed with the primer pair targeting the first and last exon of the BARD1 transcript, and the PCR products were visualized on a 1% w/v agarose gel stained with ethidium bromide as previously described^22^.

### Quantitative real-time PCR

To quantify the expression of BARD1β, SYBR green quantitative real-time PCR reaction was performed as previously described^22^ with the following modifications. Superscript™ III First-Strand Synthesis SuperMix and Oligo(dT)20 primers by Invitrogen (Carlsbad, CA) was used for cDNA synthesis. The expression of BARD1β was normalized to GAPDH and the results were analyzed by the comparative Ct method. Quantitative PCR was performed at least three times from three different RNA extractions for each cell line. Statistical comparisons were performed on mean expression of the independent samples and fold differences among colon cancer cell lines were calculated.

### Cell cycle analysis

The cell cycle distribution of stable SW480 cells was analyzed by using propidium iodide (PI) staining. Briefly, 10^6^ cells were trypsinized and washed with PBS, centrifuged and fixed in ice-cold 70% ethanol and washed with cold PBS, and supernatant was removed. For measurement of DNA content, cells were washed with PBS and then stained with 100 μg/mL RNase A in PBS (Sigma), 50 μg/mL PI (Sigma) and 0.1% (v/v) Triton X-100 for 30 min at room temperature in dark, and analyzed by flow cytometry (Becton-Dickinson, Franklin Lakes, NJ, USA) with Cell Quest software. Three independent experiments were performed for each cell line.

### Gene knockdown

We conducted shRNA-mediated knockdown of BARD1β gene in Caco-2 cells and studied the effects on cell viability. The BARD1b target sequence was: (5′-GCTAGCCACTGCTCAGTAATG). We constructed short hairpin RNA (shRNA) oligos against BARD1β and cloned into pLKO.1 puro lentiviral backbone according to Addgene’s pLKO.1 protocol. Lentiviral particles were generated in 293T cells and transduced into Caco-2cells.

### Statistical analysis

All data presented as mean +/− SD. One-way ANOVA and Tukey’s multiple comparison test were used as appropriate comparison among groups. The criterion for statistical significance was set at *p < 0.05 or **p < 0.01.

## Additional Information

**How to cite this article**: Ozden, O. *et al*. Expression of an Oncogenic BARD1 Splice Variant Impairs Homologous Recombination and Predicts Response to PARP-1 Inhibitor Therapy in Colon Cancer. *Sci. Rep.* **6**, 26273; doi: 10.1038/srep26273 (2016).

## Supplementary Material

Supplementary Information

## Figures and Tables

**Table 1:** Colon cell lines listed with genetic backgrounds.

**Cell line**	**Characteristic**
FET	Early stage CRC, well diff., high TGFβ1
HCT116 p21^−/−^	p21 negative cell line HCT116
HCT116	Mismatch repair deficiency
SW480	SMAD4 null
SW620	SW480 lymph node metastatic derivative
Caco-2	p21 and SMAD4 wild type, MS stable

## References

[b1] Situ B. C. I. Cancer Facts & Figures 2015. American Cancer Society. Available at: http://www.cancer.org/acs/groups/content/@editorial/documents/document/acspc-044552.pdf (Accessed: 24th March 2016).

[b2] Weitz J. . Colorectal cancer. Lancet 365, 153–165 (2005). 15639298 10.1016/S0140-6736(05)17706-X

[b3] SEER Cancer Statistics Factsheets: *Colon and Rectum Cancer. National Cancer Institute*. Bethesda, MD. Available at: http://seer.cancer.gov/statfacts/html/colorect.html (Accessed: 24th March 2016).

[b4] Tennstedt P. . RAD51 overexpression is a negative prognostic marker for colorectal adenocarcinoma. Int J Cancer 132, 2118–2126 (2013). 23065657 10.1002/ijc.27907

[b5] Ohta T., Sato K. & Wu W. W. The BRCA1 ubiquitin ligase and homologous recombination repair. Febs Letters 585, 2836–2844 (2011). 21570976 10.1016/j.febslet.2011.05.005

[b6] Shabbeer S. . BRCA1 targets G2/M cell cycle proteins for ubiquitination and proteasomal degradation. Oncogene 32, 5005–5016 (2013). 23246971 10.1038/onc.2012.522 PMC3796024

[b7] Wu L. J. C. . Identification of a RING protein that can interact *in vivo* with the BRCA1 gene product. Nature Genetics 14, 430–440 (1996). 8944023 10.1038/ng1296-430

[b8] Hashizume R. . The RING heterodimer BRCA1-BARD1 is a ubiquitin ligase inactivated by a breast cancer-derived mutation. Journal of Biological Chemistry 276, 14537–14540 (2001). 11278247 10.1074/jbc.C000881200

[b9] Irminger-Finger I. & Jefford C. E. Is there more to BARD1 than BRCA1? Nature Reviews Cancer 6, 382–391 (2006). 16633366 10.1038/nrc1878

[b10] Miki Y. . A Strong Candidate for the Breast and Ovarian-Cancer Susceptibility Gene Brca1. Science 266, 66–71 (1994). 7545954 10.1126/science.7545954

[b11] Savage K. I. & Harkin D. P. BRCA1, a ‘complex’ protein involved in the maintenance of genomic stability. Febs J 282, 630–646 (2014). 25400280 10.1111/febs.13150

[b12] Ghimenti C. . Germline mutations of the BRICA1-associated ring domain (BARD1) gene in breast and breast/ovarian families negative for BRCA1 and BRCA2 alterations. Genes Chromosomes & Cancer 33, 235–242 (2002). 11807980 10.1002/gcc.1223

[b13] Phelan C. M. . Incidence of colorectal cancer in BRCA1 and BRCA2 mutation carriers: results from a follow-up study. Br J Cancer 110, 530–534 (2014). 24292448 10.1038/bjc.2013.741 PMC3899769

[b14] Liu C. . A fine-scale dissection of the DNA double-strand break repair machinery and its implications for breast cancer therapy. Nucleic Acids Res 42, 6106–6127 (2014). 24792170 10.1093/nar/gku284 PMC4041457

[b15] Bryant H. E. . Specific killing of BRCA2-deficient tumours with inhibitors of poly(ADP-ribose) polymerase. Nature 434, 913–917 (2005). 15829966 10.1038/nature03443

[b16] Farmer H. . Targeting the DNA repair defect in BRCA mutant cells as a therapeutic strategy. Nature 434, 917–921 (2005). 15829967 10.1038/nature03445

[b17] Fong P. C. . Inhibition of poly(ADP-ribose) polymerase in tumors from BRCA mutation carriers. N Engl J Med 361, 123–134 (2009). 19553641 10.1056/NEJMoa0900212

[b18] Arnaudeau C., Lundin C. & Helleday T. DNA double-strand breaks associated with replication forks are predominantly repaired by homologous recombination involving an exchange mechanism in mammalian cells. Journal of Molecular Biology 307, 1235–1245 (2001). 11292338 10.1006/jmbi.2001.4564

[b19] Li M. & Yu X. C. Function of BRCA1 in the DNA Damage Response Is Mediated by ADP-Ribosylation. Cancer Cell 23, 693–704 (2013). 23680151 10.1016/j.ccr.2013.03.025 PMC3759356

[b20] Hu Y. D. . PARP1-Driven Poly-ADP-Ribosylation Regulates BRCA1 Function in Homologous Recombination-Mediated DNA Repair. Cancer Discovery 4, 1430–1447 (2014). 25252691 10.1158/2159-8290.CD-13-0891 PMC4258125

[b21] Kleiman F. E. . BRCA1/BARD1 inhibition of mRNA 3′ processing involves targeted degradation of RNA polymerase II. Genes Dev 19, 1227–1237 (2005). 15905410 10.1101/gad.1309505 PMC1132008

[b22] Sporn J. C., Hothorn T. & Jung B. BARD1 Expression Predicts Outcome in Colon Cancer. Clinical Cancer Research 17, 5451–5462 (2011). 21693656 10.1158/1078-0432.CCR-11-0263 PMC3372413

[b23] Zhang Y. Q. . Expression of oncogenic BARD1 isoforms affects colon cancer progression and correlates with clinical outcome. British Journal of Cancer 107, 675–683 (2012). 22814582 10.1038/bjc.2012.297 PMC3419952

[b24] Bosse K. R. . Common Variation at BARD1 Results in the Expression of an Oncogenic Isoform That Influences Neuroblastoma Susceptibility and Oncogenicity. Cancer Research 72, 2068–2078 (2012). 22350409 10.1158/0008-5472.CAN-11-3703 PMC3328617

[b25] Li L. . Oncogenic BARD1 isoforms expressed in gynecological cancers. Cancer Research 67, 11876–11885 (2007). 18089818 10.1158/0008-5472.CAN-07-2370

[b26] Lord C. J. & Ashworth A. Mechanisms of resistance to therapies targeting BRCA-mutant cancers. Nat Med 19, 1381–1388 (2013). 24202391 10.1038/nm.3369

[b27] McGlynn P. & Lloyd R. G. Recombinational repair and restart of damaged replication forks. Nat Rev Mol Cell Biol 3, 859–870 (2002). 12415303 10.1038/nrm951

[b28] Fabbro M., Rodriguez J. A., Baer R. & Henderson B. R. BARD1 induces BRCA1 intranuclear foci formation by increasing RING-dependent BRCA1 nuclear import and inhibiting BRCA1 nuclear export. J Biol Chem 277, 21315–21324 (2002). 11925436 10.1074/jbc.M200769200

[b29] Chen A., Kleiman F. E., Manley J. L., Ouchi T. & Pan Z. Q. Autoubiquitination of the BRCA1-BARD1 RING ubiquitin ligase. Journal of Biological Chemistry 277, 22085–22092 (2002). 11927591 10.1074/jbc.M201252200

[b30] Jefford C., Feki A., Harb J., Irminger-Finger E. T. & Krause K. Characterization of BARD1 protein regions correlated with sub-cellular localization and required for apoptotic activity. Mol Biol Cell 13, 306a–306a (2002).

[b31] Jefford C. E., Feki A., Harb J., Krause K. H. & Irminger-Finger I. Nuclear-cytoplasmic translocation of BARD1 is linked to its apoptotic activity. Oncogene 23, 3509–3520 (2004). 15077185 10.1038/sj.onc.1207427

[b32] Franken N. A. P., Rodermond H. M., Stap J., Haveman J. & van Bree C. Clonogenic assay of cells *in vitro*. Nature Protocols 1, 2315–2319 (2006). 17406473 10.1038/nprot.2006.339

[b33] Rao G. H. . Establishment of a human colorectal cancer cell line P6C with stem cell properties and resistance to chemotherapeutic drugs. Acta Pharmacol Sin 34, 793–804 (2013). 23736004 10.1038/aps.2013.56 PMC3674520

[b34] Chen J. & Weiss W. A. Alternative splicing in cancer: implications for biology and therapy. Oncogene 34, 1–14 (2015). 24441040 10.1038/onc.2013.570

[b35] McCarthy E. E., Celebi J. T., Baer R. & Ludwig T. Loss of Bard1, the heterodimeric partner of the Brca1 tumor suppressor, results in early embryonic lethality and chromosomal instability. Molecular and Cellular Biology 23, 5056–5063 (2003). 12832489 10.1128/MCB.23.14.5056-5063.2003 PMC162231

[b36] Mukhopadhyay A. . Development of a functional assay for homologous recombination status in primary cultures of epithelial ovarian tumor and correlation with sensitivity to poly(ADP-ribose) polymerase inhibitors. Clin Cancer Res 16, 2344–2351 (2010). 20371688 10.1158/1078-0432.CCR-09-2758

[b37] Shah M. M. . An *ex vivo* assay of XRT-induced Rad51 foci formation predicts response to PARP-inhibition in ovarian cancer. Gynecol Oncol 134, 331–337 (2014). 24844596 10.1016/j.ygyno.2014.05.009 PMC4221419

[b38] Ryser S. . Distinct Roles of BARD1 Isoforms in Mitosis: Full-Length BARD1 Mediates Aurora B Degradation, Cancer-Associated BARD1 beta Scaffolds Aurora B and BRCA2. Cancer Research 69, 1125–1134 (2009). 19176389 10.1158/0008-5472.CAN-08-2134

[b39] Wang M. . PARP-1 and Ku compete for repair of DNA double strand breaks by distinct NHEJ pathways. Nucleic Acids Res 34, 6170–6182 (2006). 17088286 10.1093/nar/gkl840 PMC1693894

[b40] Sharma S. . Homology and enzymatic requirements of microhomology-dependent alternative end joining. Cell Death Dis 6, e1697 (2015). 25789972 10.1038/cddis.2015.58 PMC4385936

[b41] Lamouille S., Xu J. & Derynck R. Molecular mechanisms of epithelial-mesenchymal transition. Nat Rev Mol Cell Bio 15, 178–196 (2014). 24556840 10.1038/nrm3758 PMC4240281

[b42] Morin P. J. . Activation of beta-catenin-Tcf signaling in colon cancer by mutations in beta-catenin or APC. Science 275, 1787–1790 (1997). 9065402 10.1126/science.275.5307.1787

[b43] Lee S. A., Roques C., Magwood A. C., Masson J. Y. & Baker M. D. Recovery of deficient homologous recombination in Brca2-depleted mouse cells by wild-type Rad51 expression. DNA Repair (Amst) 8, 170–181 (2009). 18992372 10.1016/j.dnarep.2008.10.002

[b44] Paull T. T. . A critical role for histone H2AX in recruitment of repair factors to nuclear foci after DNA damage. Curr Biol 10, 886–895 (2000). 10959836 10.1016/s0960-9822(00)00610-2

[b45] Bauer J., Sporn J. C., Cabral J., Gomez J. & Jung B. Effects of activin and TGFbeta on p21 in colon cancer. Plos One 7, e39381 (2012). 22761777 10.1371/journal.pone.0039381 PMC3383701

[b46] Dizin E. & Irminger-Finger I. Negative feedback loop of BRCA1-BARD1 ubiquitin ligase on estrogen receptor alpha stability and activity antagonized by cancer-associated isoform of BARD1. Int J Biochem Cell Biol 42, 693–700 (2010). 20060929 10.1016/j.biocel.2009.12.025

