# Supplementary material for: Expression of an Oncogenic BARD1 Splice Variant Impairs Homologous Recombination and Predicts Response to PARP-1 Inhibitor Therapy in Colon Cancer
Source: Sci Rep. 2016 May 20;6:26273. doi: 10.1038/srep26273 (PMC4873788; doi:10.1038/srep26273)
Supplement: Supplementary Information [file srep26273-s1.pdf]

Supplementary Information

Expression of an Oncogenic BARD1 Splice Variant Impairs Homologous Recombination and Predicts Response to PARP-1 Inhibitor Therapy in Colon Cancer

Ozkan Ozden, Faraz Bishehsari, Jessica Bauer, Seong-Hoon Park, Arundhati Jana, Seung Hyun Baik, Judith C. Sporn, Jonas J. Staudacher, Cemal Yazici, Nancy Krett, and Barbara Jung

Supplementary Section Inventory:

Supplementary Figure S1 relates to manuscript figure 3c
Supplementary Figure S2 relates to manuscript figure 3e
Supplementary Figure S3 relates to manuscript figure 6a

Supplementary Data

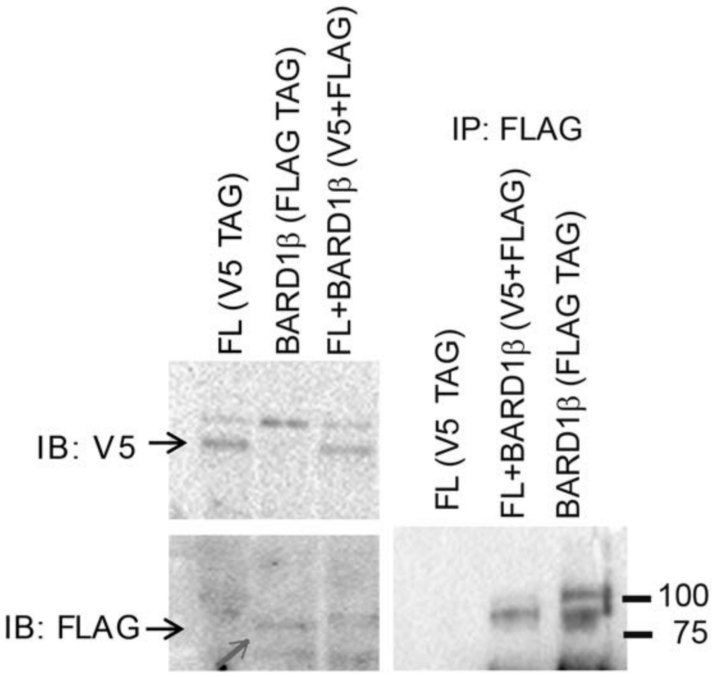

Supplemental Figure S1: Exogenous expression of SV BARD1β in SW480 cells. SW480 cells overexpressing FLAG-tagged BARD1β, V5-tagged full length (FL) BARD1, or both BARD1β and FL were generated using lentivirus infection.

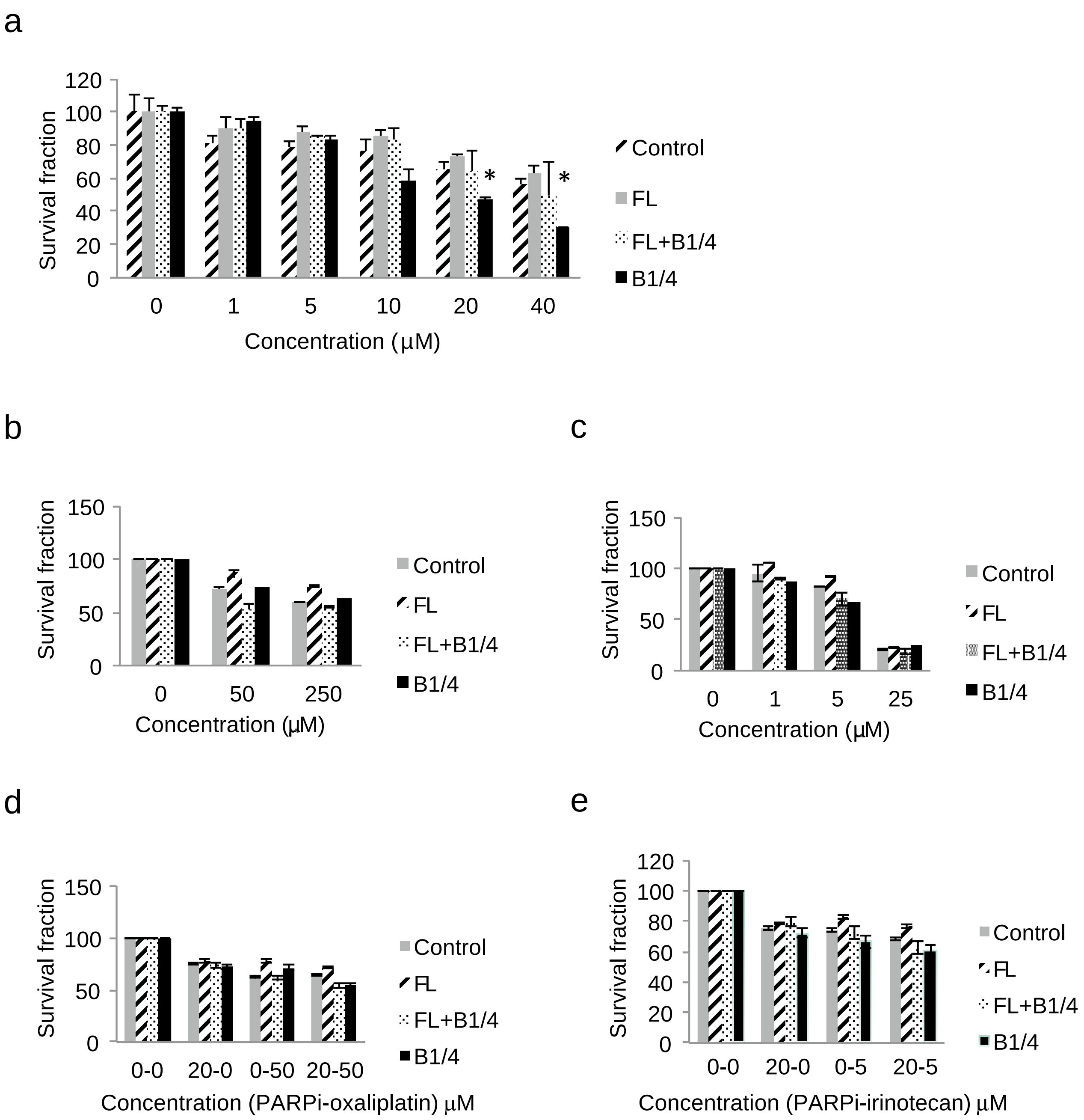

Supplemental Figure S2: Exogenous expression of SV BARD1b in SW480 cells imparts sensitivity to PARP inhibition. SW480 cells stably infected with either full length BARD1 (FL), BARD1β, or the combination FL + BARD1β, were exposed to either PARPi for 96 hours (a), only oxaliplatin for 36 hours (b), only irinotecan for 36 hours (c), both PARPi and oxaliplatin for 36 hours (d), or both PARPi and irinotecan for 36 hours (e). Survival fraction was determined using cell viability assays. (*$p < 0.01$ relative to control).

a

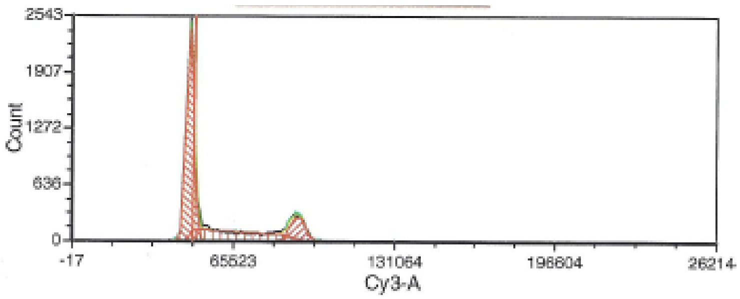

b

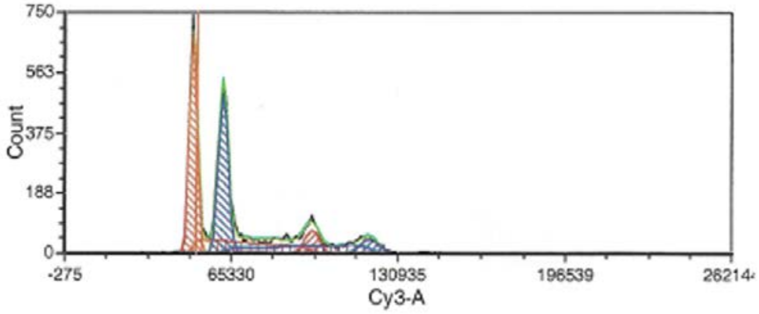

c

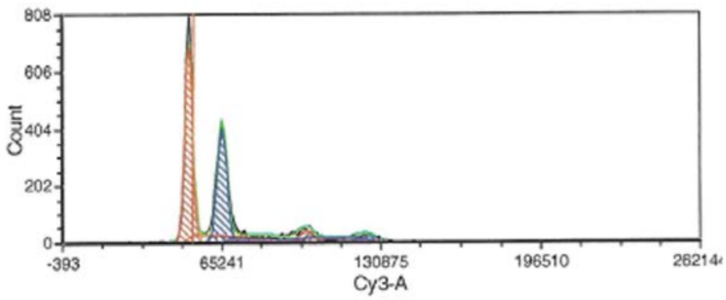

d

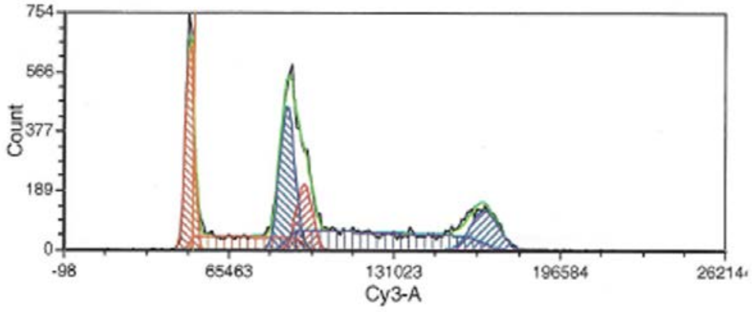

Supplemental Figure S3: DNA histograms for cell cycle analysis. Control (a), FL (b), FL+BARD1β (c), and BARD1β (d) expressing SW480 cells were stained with propidium iodide (PI), and monitored by flow cytometry to determine the percentage of G1 phase cells. Experiments were repeated at least three times. (*$p < 0.01$ relative to control).
